# Obituary GIDEON J. MELLENBERGH (1938–2021)

**DOI:** 10.1007/s11336-021-09784-z

**Published:** 2021-07-13

**Authors:** Denny Borsboom, Henk Kelderman, Wim J. van der Linden

**Affiliations:** 1 University of Amsterdam, Amsterdam, Netherlands; 2 Leiden University, Leiden, Netherlands; 3 University of Twente, Enschede, Netherlands

Gideon Jan Mellenbergh (known as Don to his friends and colleagues) was born in Amsterdam on August 9, 1938, and passed away in the same city on March 27, 2021. Don was a major force in the Dutch psychometric community. He was one of the founders of the Interuniversity Graduate School of Psychometrics and Sociometrics (IOPS) that united the Ph.D. programs in the Netherlands and Flanders, and which he directed between 1987 and 2000. He was a member of the Royal Dutch Academy of Sciences (KNAW), served on the boards of the Netherlands Organization for Scientific Research and National Institute of Educational Measurement, and presided accreditation committees to evaluate Dutch and Flemish university programs. Don was a connecting figure who was able to build and maintain good relationships with all members of the Dutch psychometric community, even where there were passionate differences of opinion about psychometrics. He did not care for schools of thought; he only distinguished between good and bad research—and disliked the latter. This motivated a lifelong quest to improve research methods in psychology.

Don attended *Hervormd Gymnasium* in Amsterdam, a six-year secondary school for gifted students with a curriculum consisting of six mandatory languages (including Greek and Latin), from which he graduated in 1957 with a specialization in the natural sciences and mathematics. After shortly considering to enroll in the college of physical education—Don was an accomplished basketball player—he attended the six-year psychology program at the University of Amsterdam. Don was not very impressed with the curriculum until he met Adriaan de Groot, a towering figure in postwar Dutch psychology and the founder of the university’s Psychological Methods group. After his graduation in 1965, Don was appointed as Assistant Professor of Psychological Methods. His first job was at the Department of Exam Techniques, a precursor of the Dutch educational testing bureau Cito (which was also founded by De Groot). This department was headed by Robert van Naerssen, a psychometrician far ahead of his time (van den Brink & Mellenbergh, 1984), whose impact was, however, limited as he published mainly in Dutch. Van Naerssen influenced Don’s thinking greatly, and Don would commemorate his ideas and contributions often in conversation. Don defended his Ph.D. thesis, entitled *Studies on Educational Tests, *in 1971. The dissertation, in which he reported psychometric analyses of the *Amsterdam School Test*, contains the first documented operational use of the Rasch model in The Netherlands.

The next step in his academic career was an appointment as an Associate Professor in the Psychology program at Utrecht University in 1971, with the assignment to develop a new specialization in psychometrics. The appointment turned out to be a disappointment. One of the oddities he faced was a just transformed academic council that now included all faculty and students operating on a one-man one-vote basis. The council rejected Don’s proposal for his new program because it was “too directive” according to a dominant Marxist faction (among other objections, the faction challenged the idea that professors had the authority to decide which answers to exam questions were correct). As the academic environment proved unworkable, Don decided to accept a demotion to return to his old position at Amsterdam. He did so with a bang: upon his departure, he wrote a widely distributed pamphlet lamenting the deplorable state of the department. The main problem among faculty and students, Don argued, was an addiction to “fundamental discussion.” Or in his own words: “You first have to discuss your educational objectives fundamentally before you’re allowed to draft a study program; you first have to discuss the position of psychology in society fundamentally before you’re allowed to do any research; you first have to scrutinize your own motivation before you’re allowed to open a book on psychology. Continuing the argument, you’ll first have to confirm that humans descend from apes before you’re allowed to eat any bananas” (Mellenbergh, 1975).

The pamphlet hit the university administration as a bomb, was discussed in Dutch parliament, and became an important piece in promoting the no-nonsense philosophy that would gain influence in Dutch academia in the next decades. Don strongly adhered to this no-nonsense philosophy. As the son of a taxi driver, he was one of the early go-getters that gained access to quality education and the elitist bastion of the academy. In fact, one of his main complaints about the Marxist factions in Utrecht was that he found them precisely as culturally elitist as the authorities they challenged. Like so many of the socially mobile, Don was averse to nepotism and judged people solely on their potential and merit; importantly, he showed the way to many students of similar backgrounds to his own. Educational politics never ceased to interest Don, and a few months before his passing away, he completed a book manuscript with all his reflections on a history of the developments during his academic career (Mellenbergh, in preparation).

Back in Amsterdam, Don’s star rose quickly. In 1982, he was offered the Chair of Psychological Methods, which had become vacant upon the retirement of Fred Kerlinger. It was remarkable that he got this appointment because the appointment of a previous candidate was blocked because he had done research for NATO. It later turned out that Don had committed the same sin, but miraculously escaped cancellation. Don led the program group of Psychological Methods until 1999 and mandatorily retired in 2003. In fact, except for his disappointment at Utrecht, the only interruptions in his career at Amsterdam were visiting professorships at the University of Chicago (1969) and University of Santiago de Compostella, Spain (1991, 1993). His abundant love for Amsterdam was not parochial though. For instance, to prepare his visits to Spain, he started learning Spanish and quickly achieved mastery of the language at a level that allowed him to lecture in Spanish.

Don had a tremendous academic offspring, and much of his influence was via his students. During his career, he was the mentor of 89 Ph.D. candidates with a completed dissertation, a stunning number that few if any psychologists working at the time could match. Of these, 43 became full professors, and no less than seven obtained a chair in research methods. Don often commemorated the adage “if it isn’t published, it hasn’t happened,” and accordingly, he was one of the instigators of transitioning from a practice in which dissertations were books (often unpublished) to the current tradition in which the core of a dissertation is formed by a collection of some five manuscripts which are already submitted for publication or published. In total, Don thus has mentored over 400 of those manuscripts. Through his many mentorships of influential psychometricians and psychologists, Don was a major force operating in the background of twentieth-century psychology; many of his students helped to change the face of social and behavioral science. In addition, psychometricians passed on his psychometric ideas years to hundreds of bachelor students in The Netherlands through the textbook Test theory and test construction, which he wrote together with Wulfert van den Brink (van den Brink & Mellenbergh, 1998).

Many Dutch psychometricians remember Don’s reading groups during which books on general topics relevant to psychometrics were studied. One particularly important reading group studied the seminal work by Bishop, Fienberg, and Holland (1975/2007). This led to the first papers on log-linear item response theory (Mellenbergh & Vijn, 1981), which in turn opened the door to a plethora of extensions and applications. One of these applications involved the first articulations of DIF in a form that has now become the standard—as a conditional dependence problem—and the introduction of the well-known concepts of uniform and non-uniform DIF (Mellenbergh, 1982, 1985, 1989). Having been one of the first to recognize the practical power of item response theory, his next interest was in the application of statistical decision theory to educational testing, specifically its potential to solve the problems of culture fair selection and pass–fail decisions in education (Mellenbergh et al., Mellenbergh et al. (1977); Mellenbergh & van der Linden, 1979, 1981; Mellenbergh, 1982; van der Linden & Mellenbergh, 1976, 1977).

Less well known is Don’s settlement of a terminological issue that had plagued the early psychometric literature. Until the mid-1990s, it was common to see response models for items with multiple answer categories referenced to as “polychotomous.” When David Weiss, then editor of Applied Psychological Measurement, began planning a special issue on these models, he felt the need to standardize (Weiss, 1995). He consulted Don Mellenbergh who, thanks to his training in classical Greek, was able to tell him that “dichotomous” is a contraction of “dicho” (two) and “tomous” (a cut), whereas “polytomous” is the contraction of “poly” (many) and “tomous.” David Weiss accepted his explanation, standardized the language in the special issue, and ever since the use of “polychotomous” has disappeared from the literature.

Don was a generalist who typically aimed to characterize the essence of models and techniques. In the first decades of postwar psychometrics, many different latent variable models had been formulated (e.g., the IRT model, the factor model, the latent class model, etc.), often using different terminology and notation. Don was one of the first to realize that many different latent variable models could be formulated in the same framework, namely as generalized linear models where the linear predictor contained latent variables. This led to one of his most important publications, which introduced the generalized linear item response theory (GLIRT) model (Mellenbergh, 1994a). This model subsumed most of the known measurement models, including models for continuous responses, and bridged the divide between factor analysis and item response theory, so that ideas from item response theory, such as measurement precision (Mellenbergh, 1996) and specific objectivity (Mellenbergh, 1994b), could also be applied to factor analytic models. The idea that all psychometric models basically express the same conceptual hypothesis—namely that the statistical association between different items arises from their common dependence on a latent variable—later led to conceptual investigations into the common theoretical status of latent variable models (Borsboom, Mellenbergh, & Van Heerden, 2003).

One of the leading ideas in Don’s psychometric philosophy was that Item Response Theory should be seen as a theory that describes how item response patterns arise, not just as a statistical model that summarizes the probability distribution of these item response patterns. In particular, Don viewed IRT as a theory of item response behavior (he often referred to this as a “mini-theory”). As a consequence, understanding item response processes is essential to the analysis of test validity: for if one does not understand how item responses arise, one cannot understand what a test measures. This idea underwrites the articulation of validity as a causal concept, which depends crucially on the processes that transfer differences in a measured attribute into differences in item response patterns (Borsboom, Mellenbergh, & Van Heerden, 2004).

When asked why Don was so remarkably successful, a colleague once remarked: “Well, basically he has two hobbies. One is reading papers, and the other is writing them.” And indeed, after his retirement in 2003, instead of withdrawing from academic life, Don started pursuing new topics with the vigor of a young man. Most important among these was the topic of statistical and methodological consultancy. With Herman Adér, Don developed and taught the first courses on this topic, and wrote a book to be used in education of methodologists (Adér & Mellenbergh, 2008). Since, consultancy has become a standard element in the education of methodologists and psychometricians in The Netherlands. His next project was a new textbook of nearly 500 pages with a conceptual introduction to psychometrics (Mellenbergh, 2011). The final years of Don’s life were devoted to a book with the working title *Against Error* (a reference to Feyerabend’s *Against Method*), which was eventually published as *Counteracting methodological errors in behavioral research*, no less than sixteen years after his retirement (Mellenbergh, 2019).

Don’s friends and colleagues remember him as an inspiration, a person who always gave and never profited from others, and a psychometrician who left an enormous legacy. But, above all, we remember him with deep gratitude and respect.

*Denny Borsboom, Henk Kelderman, and Wim J. van der Linden*

## References

[CR1] Adér, H. J. & Mellenbergh, G. J. (with contributions by D. J. Hand). (2008). *Advising on research methods*. Huizen: Van Kessel.

[CR2] Bishop, Y. M., Fienberg, S. E., & Holland, P. W. (2007). Discrete multivariate analysis: Theory and practice. Springer (**Original work published 1975**).

[CR3] Borsboom D, Mellenbergh GJ, Van Heerden J (2003). The theoretical status of latent variables. Psychological Review.

[CR4] Borsboom D, Mellenbergh GJ, Van Heerden J (2004). The concept of validity. Psychological Review.

[CR5] Mellenbergh GJ (1975). De verloedering ener subfaculteit [The degeneration of a subfaculty]. Wetenschap en Democratie.

[CR6] Mellenbergh GJ (1982). Contingency table models for assessing item bias. Journal of Educational Statistics.

[CR7] Mellenbergh GJ (1985). Vraag-onzuiverheid: Definitie, detectie en onderzoek. [Item bias: definition, detection, and research]. Nederlands Tijdschrift voor de Psychologie.

[CR8] Mellenbergh GJ (1989). Item bias and item response theory. International Journal of Educational Research.

[CR9] Mellenbergh GJ (1994). Generalized linear item response theory. Psychological Bulletin.

[CR10] Mellenbergh GJ (1994). A unidimensional latent trait model for continuous item responses. Multivariate Behavioral Research.

[CR11] Mellenbergh GJ (1996). Measurement precision in test score and item response models. Psychological Methods.

[CR12] Mellenbergh GJ (2011). A conceptual introduction to psychometrics.

[CR13] Mellenbergh GJ (2019). Counteracting methodological errors in behavioral research.

[CR14] Mellenbergh, G. J. (in preparation). *Universitaire Metamorfosen [University Metamorphoses]*.

[CR15] Mellenbergh GJ, Koppelaar H, van der Linden WJ (1977). Dichotomous decisions based on dichotomously scored test items: A case study. Statistica Neerlandica.

[CR16] Mellenbergh GJ, van der Linden WJ (1979). The internal and external optimality of decisions based on tests. Applied Psychological Measurement.

[CR17] Mellenbergh GJ, van der Linden WJ (1981). The linear utility model for optimal selection. Psychometrika.

[CR18] Mellenbergh GJ, Vijn P (1981). The Rasch model as a loglinear model. Applied Psychological Measurement.

[CR19] van den Brink, W.P., & Mellenbergh, G.J. (1984). Robert F. van Naerssen: Psychometricus [Robert F. van Naerssen: Psychometrician]. *Tijdschrift voor Onderwijsresearch,**9*.

[CR20] van den Brink WP, Mellenbergh GJ (1998). Testleer en testconstructie [Test theory and test construction].

[CR21] van der Linden WJ, Mellenbergh GJ (1976). Optimal cutting scores using a linear loss functions. Applied Psychological Measurement.

[CR22] van der Linden WJ, Mellenbergh GJ (1977). Coefficients for tests from a decision theoretic point of view. Applied Psychological Measurement.

[CR23] Weiss DJ (1995). Polychotomous or polytomous?. Applied Psychological Measurement.

